# Patient‐reported and performance‐based measures of walking in mild–moderate Parkinson's disease

**DOI:** 10.1002/brb3.1081

**Published:** 2018-08-22

**Authors:** Breiffni Leavy, Niklas Löfgren, Maria Nilsson, Erika Franzén

**Affiliations:** ^1^ Department of Neurobiology, Care Sciences and Society Division of Physiotherapy Karolinska Institutet Huddinge Sweden; ^2^ Stockholms Sjukhem Foundation Stockholm Sweden; ^3^ Function Area Occupational Therapy & Physiotherapy, Allied Health Professionals Function Karolinska University Hospital Stockholm Sweden; ^4^ Department of Health Sciences Lund University Lund Sweden; ^5^ Memory Clinic Skåne University Hospital Malmö Sweden

**Keywords:** outcome assessment, Parkinson's disease, walking

## Abstract

**Background:**

Knowledge of the relationships between patient‐reported and performance‐based walking measures in Parkinson's disease (PD) should inform clinical decision‐making. The Walk‐12G reliably captures perceived walking difficulties but has not been compared to performance‐based walking in laboratory or free‐living settings or across different groups.

**Objectives:**

To investigate the relationship between patient‐reported walking difficulties (Walk‐12G) and performance‐based walking in laboratory and free‐living conditions and to determine whether the Walk‐12G can distinguish between the subgroups, (i) people with/without PD and (ii) mild/moderate disease stages.

**Methods:**

Forty‐seven people without and 49 people with PD (Hoehn and Yahr stage II and III) were assessed in relation to patient‐reported walking difficulties (Walk‐12G scale); spatiotemporal gait characteristics (Pace; Rhythm; Asymmetry; Variability; and Postural control) using a laboratory‐based electronic walkway; and walking behavior (mean steps/day and minutes of brisk walking/day) using accelerometers in free‐living conditions.

**Results:**

The Walk‐12G correlated moderately with the spatiotemporal domain step velocity (*r *= −0.46) and walking behavior, measured as mean steps/day (*r *= −0.46). Weaker correlations were observed for step length and minutes spent in brisk walking (*r *= −0.36 and *r *= −0.35, respectively). Poor correlations were observed for all other spatiotemporal domains. The Walk‐12G could distinguish between people with and without PD (Effect size, *r* = 0.82) and between those at mild/moderate disease stages (*r* = 0.34).

**Conclusions:**

Perceived walking difficulties showed weak to moderate associations with performance‐based measures of walking in mild–moderate PD. As the strongest associations were observed for step velocity and walking behavior, targeting these specific gait aspects could improve perceived walking difficulties in daily life.

## 1 INTRODUCTION

Impairments in gait manifest in the early disease stages of Parkinson's disease (PD) and gradually increase in line with disease progression (Galna, Lord, Burn, & Rochester, 2015; Hausdorff, 2009). PD‐specific gait impairments, such as shorter, more variable, and shuffling steps, can lead to a negative spiral of activity limitations, physical inactivity, and muscle weakness, which further predisposes this group to falls and related injuries (Allen, Schwarzel, & Canning, 2013; Lord, Godfrey et al., 2013; van Nimwegen et al., 2011). Additionally, impaired gait negatively affects a person's social participation, which can further reduce quality of life (Hammarlund, Andersson, Andersson, Nilsson, & Hagell, 2014). Gait training is therefore an integral approach within PD rehabilitation, and there is strong evidence that improvements in walking can be maintained up to 6 months after training has ceased (Mak, Wong‐Yu, Shen, & Chung, 2017). Assessment of walking is not only an essential marker of rehabilitation effectiveness, but can also potentially identify those at risk for physical inactivity or falls who require targeted preventive efforts (Canning, Paul, & Nieuwboer, 2014).

Walking is a multidimensional activity that can be assessed using a range of clinical outcome assessments. These assessments are selected to measure a specific concept of interest and can be classified according to the person or means by which judgment affected the measurement; patient‐reported; clinician‐reported; observer‐reported; or performance outcomes (Walton et al., 2015).

Performance outcomes at the microlevel of gait assessment include spatiotemporal characteristics, which are reliably captured using electronic walkways (Godinho et al., 2016). A recent gait model, validated on a PD population, found five independent domains to represent the overarching construct of gait—Pace; Rhythm; Variability; Asymmetry; and Postural control (Lord, Galna, & Rochester, 2013). Whereas the PD‐specific symptoms, bradykinesia and rigidity, contribute to disturbances in the Pace and Rhythm, the unilateral debut of these symptoms manifests as increased gait Asymmetry (Lord, Galna, Verghese et al., 2013; Peterson & Horak, 2016). Gait Variability increases in line with disease progression and may have the potential to predict falls in PD (Hausdorff, 2005, 2007). Impaired Postural control is seen in the size of voluntary and reactive stepping responses and can be measured using step width (Peterson & Horak, 2016). According to the International Classification of Functioning, Disability and Health, gait analysis using electronic walkways represents measures of walking capacity in a standardized test situation. However, laboratory‐based gait assessments are often conducted during highly controlled circumstances in a specific situation and may not represent actual walking behavior, thereby limiting the ecological validity. Instead, walking behavior in everyday life can be measured using accelerometers, which are wearable devices measuring body acceleration during a specific wear‐time period (Matthews, Hagstromer, Pober, & Bowles, 2012). Measuring walking in everyday life is especially relevant in PD as this group are less physically active than people without the disease (van Nimwegen et al., 2011). While such objective gait assessments provide important information, they are not feasible in most clinical settings. Self‐reported measures, on the other hand, are easily accessible and provide important complementary information. As individual perception of one's abilities in a specific situation is likely to influence actual behavior, it appears highly relevant to investigate how this relates to actual abilities, particularly regarding waking in individuals with PD. Indeed, due to the progressive nature of PD, it is vital to remain physically active, while at the same time be conscious about limitations in order to avoid injuries. Indeed, excessive risk‐taking has been identified as a particular risk factor for falls in individuals with PD (Smulders, Esselink, Cools, & Bloem, 2014).

To achieve collaborative patient‐centered rehabilitation in PD, it is necessary to target and assess training which is responsive to patient preferences. Therefore, when assessing walking, performance measures should be complemented with patient‐reported measures reflecting perceived difficulty in everyday life (van der Eijk, Nijhuis, Faber, & Bloem, 2013). The generic walking scale (Walk‐12G) is a patient‐reported measure of walking difficulties in 12 everyday situations. It stems initially from a Multiple Sclerosis Walking questionnaire (Hobart, Riazi, Lamping, Fitzpatrick, & Thompson, 2003), was adapted for other neurological conditions (Holland, O'Connor, Thompson, Playford, & Hobart, 2006) and then into a non‐disease‐specific version which is available in Swedish (Bladh et al., 2012). The Walk‐12G is quick to complete and shows good data completeness and high test−retest reliability in PD, which further motivates its clinical application (Bladh et al., 2012). Although moderate correlations are reported between the Walk‐12G and clinical assessments of mobility and gait speed in a small sample of people with PD (Bladh et al., 2012), no previous study has tested the extent to which the scale relates to capacity or behavior‐based measures of walking in controlled and free‐living environments. Such knowledge would highlight the extent to which objective performance‐based measures are in line with how people with PD perceive their walking ability. This information would in turn further enable clinicians to target those aspects of gait most strongly linked to patient‐perceived walking ability. Additionally, it has not been confirmed if the Walk‐12G can differentiate between different subgroups of individuals with established differences in walking abilities, such as people with and without PD and between people with different PD severity. This knowledge would add to the interpretability of the Walk‐12G and provide further evidence for its clinical application. This study aims to investigate the relationship between patient‐reported walking difficulties in PD (the Walk‐12G) and performance‐based measures of walking tested in laboratory and free‐living conditions. We also aimed to investigate the ability of the Walk‐12G to discriminate between healthy older adults and people with PD and between those with mild and moderate disease severity.

## 2 METHODS

### 2.1 Design

This was a preplanned cross‐sectional study whereby data collection of patient‐reported walking measures was added to the follow‐up assessments of people with PD who had previously participated in a randomized controlled trial (RCT) of a 10‐week balance training intervention (Trial number: NCT1417598). Data collection for people with PD occurred at the 9th or 12th month follow‐up of the training intervention.

### 2.2 Participants

We included 49 people with PD according to the following inclusion criteria; Neurologist diagnosed idiopathic PD (Queen Square Brain Bank Criteria); mild−moderate disease stages (stages 2−3 according to the Hoehn and Yahr scale), Mini‐Mental State Examination score ≥24 points and age ≥60 years. Exclusion criteria included coexisting neurological conditions affecting balance. All people with PD had participated in an RCT 1 year prior to the testing procedure. People without PD (*n* = 47) were recruited according to similar criteria (apart from PD diagnosis) and data collection occurred cross‐sectionally. All participants received written and verbal information about the study prior to inclusion and provided written informed consent upon inclusion. The study was approved by the regional Ethical Review Board in Stockholm, Sweden.

### 2.3 Procedure

Testing occurred during 2013−2014 at Karolinska Institutet, and commenced with an interview, followed by questionnaire administration and concluded with capacity tests to avoid performance influencing participants’ subjective reports. People with PD followed their normal scheme of medication intake and were tested during their medication ON state. Accelerometers were distributed at the end of the test sessions along with verbal and written instruction for continuous wear (apart from during bathing and sleeping) during a 7‐day period. Participants also filled in a wear‐time diary during this period.

### 2.4 Outcomes measures

#### 2.4.1 Patient‐reported walking difficulties

The Walk‐12G was self‐administered by participants at the test site. The total score ranges between 0 and 42 points, with higher scores reflecting greater perceived walking difficulties and responses refer to perceived walking difficulties during the previous 2 weeks. The first two items explore the frequency of perceived need to use support when walking indoors and outdoors, whereas the third item concerns the ability to run (items 1–3, response categories = 0−2). The remaining 9 items explore perceived difficulty regarding aspects of walking such as; exertion level; instability; distance; walking speed, and stair climbing (response categories = 0−4).

#### 2.4.2 Spatiotemporal gait characteristics in the laboratory setting

Spatiotemporal gait variables were collected in a gait laboratory during intermittent walking on a 10‐m pressure sensor mat (GAITRite; CIR Systems Inc., Franklin, NJ, USA). The GAITRite mat records each foot imprint using pressure sensors (active zone 8.3 meters) and is considered a gold standard for spatiotemporal gait assessment (Bilney, Morris, & Webster, 2003). Participants were instructed to walk at “a normal comfortable pace” and the average values for six walks was used in the analysis. To ensure a steady‐state walking speed, participants walked a distance of 3 m at both ends of the walkway to allow for acceleration and deceleration. Five independent gait domains, each consisting of two subdomains, were calculated from the GAITRite data output and included; Pace (Step velocity and Step length); Rhythm (Step time and Swing time); Variability (Step length variability and Step time variability); Asymmetry (Swing time asymmetry and Step time asymmetry); and Postural control (Step length asymmetry and Step width) (Lord, Galna, Verghese et al., 2013).

#### 2.4.3 Walking behavior in free‐living environments

Walking behavior was measured using the Actigraph GT3X+ accelerometer (Actigraph Pensacola, FL, USA) which assesses the frequency, duration, and intensity of physical activity in free‐living conditions. The accelerometer records time‐varying changes in acceleration in three planes of the axis; vertical; anteroposterior; and mediolateral. These data thresholds are previously validated using criterion measures in comparison with total energy expenditure (Sasaki, John, & Freedson, 2011) and have been tested for reliability. The outcomes mean steps per day and minutes of brisk walking (minutes of walking >1.05 m/s) per day represent walking behavior. In the calculation of these outcomes, raw acceleration data was filtered and translated into counts using the “ActiLife 6” software. Data settings were chosen using a 15‐s epoch and episodes of ≥90 min of no registered acceleration were recorded as non‐wear time and excluded from the analysis. Data from a minimum of four and maximum of 7 days was included and days where wear time was <540 min were excluded from the analysis according to recommendations (Matthews, Ainsworth, Thompson, & Bassett, 2002). Calculation of minutes of brisk walking was based on a previous calibration study among people with PD (Nero, Benka Wallen, Franzen, Stahle, & Hagstromer, 2015).

#### 2.4.4 Data analysis

Statistical analyses were performed using Stata 15.1 (StatCorp., College Station, TX, USA). The normality of the data distribution for each outcome measure was assessed using descriptive statistics and visual data inspection. Due to the skewed nature of the data Spearman's rho test was used to test the strength of the correlation between the Walk‐12G and performance‐based measures. The strength of the correlations was classified as; <0.40 = poor, 0.41–0.60 = moderate, 0.61–0.80 = good, and 0.81–1.00 = very good (Riffenburgh, 2012). Multiple comparisons were accounted for using the Bonferroni adjustment. Nonparametrical Mann–Whitney *U* tests were used to establish the between‐group differences in total Walk 12‐G score among (a) People with and without PD and (b) those at H & Y stage II and III. Effect size (ES) was calculated to estimate the magnitude of the between‐group differences. We used the following formula to calculate effect size from nonparametric tests *r* = *Z*/√*n* (Fritz, Morris, & Richler, 2012). Cohen reports the following intervals for *r*; 0.1−0.3, small effect; 03−0.5, medium effect; and 0.5 and higher, large effect (Cohen, 1988). Receiver operating characteristic (ROC) curves were calculated and areas under the curve (AUC) estimated as a test of how well the Walk‐12G performed distinguishing between different groups (people with/without PD and at those at mild/moderate disease stages) with regards to sensitivity (true positive proportion) and specificity (true negative proportion) (Swets, 1988). In terms of discriminative strength, AUC values between 0.5 and 0.7 were considered poor; 0.7 and 0.9 were considered moderate; and above 0.9 considered excellent (Hanley & McNeil, 1982). Although ROC curves are measures of diagnostic accuracy, it should be stressed that we do not consider the Walk‐12G suitable for determining the presence or absence of PD or disease severity.

## 3 RESULTS

Ninety‐six subjects performed the testing and were included in the analysis (PD, *n* = 49, people without PD, *n* = 47). Disease duration for the PD group ranged from 1.5−25 years. Demographic, mobility, and balance characteristics are outlined in Table 1. Two participants had undergone a Deep Brain Stimulation (DBS) procedure.

**Table 1:** Characteristics of all participants, *n* = 96

Demographics	People with PD (*n* = 49)		People without PD (*n* = 47)	
	Mean (*SD*)^a^	Range	Mean (*SD*)	Range
Sex (Female), *n* (%)	28 (50.9)		20 (42.5)	
Age (year)	75 (5.9)	63−89	71 (6)	60−88
Body mass index	25.7 (3.5)	17.6−32.9	23.9 (2.3)	19.6−29.6
Years with PD, median (q1–q3)^b^	6 (3−9)	1.5−26	−	−
Hoehn & Yahr stage^c^				
II, *n* (%)	(II) 22 (45)			
III, *n* (%)	(III) 27 (55)			
MMSE,^d^ median (q1–q3)	28 (27−29)	24−30	29 (27−29)	25−30
GDS,^e^ median (q1–q3)	3 (1−6)	0−12	1 (0−2)	0−7
Mobility				
Walking aid indoors, *n* (%)	4 (8)	−	0	−
Walking aid outdoors,*n* (%)	20 (41)	−	2 (4.3)	−
UPDRS motor (Part III)^f^	40 (10.9)	12−67	−	−
Physical functioning,^g^ median (q1–q3)	65 (45−80)	5−95	29 (28−30)	0−30
Freezing during walking^h^, *n* (%)				
Never/seldom	39 (79.6)			
Sometimes	5 (10.2)			
Often	5 (10.2)			
Balance and falls				
Mini‐BESTest^i^	19.8 (3.9)	10−27	22.8 (2.6)	16−27
Falls previous 12months, *n* (%)	24 (47)		8 (17)	
Falls efficacy scale‐international^j^	27.7 (8.4)	16−48	17.9 (2.1)	16−24
Patient‐reported walking difficulties				
Walk 12‐G, median (q1–q3)	12 (7‐20)			
Daily levodopa equivalent dose (mg)	635 (306)	120−1,846	−	−

Notes. PD, Parkinson's disease.

^a^ *SD*, standard deviation unless otherwise stated. ^b^q1–q3, 25th−75th percentile. ^c^Stages I–V of disease progression (I = minimal disability, V = confined to bed/wheelchair). ^d^Mini‐Mental State Examination, 0−30 (higher score = greater impairment). ^e^Geriatric Depression Scale, 0–20 (higher score = greater likelihood of depression). ^f^Motor examination of the Unified Parkinson's Disease Rating Scale, 0–108 (higher score = greater severity). ^g^Physical functioning scale of the SF‐36, 0−100 (higher score = lesser severity). ^h^Item 14 of the Unified Parkinson's Disease Rating Scale‐ Activities of daily living (UPDRS‐ADL). ^i^Mini‐Balance Evaluation Systems Test, 0–28 (higher score = better balance). ^j^Falls Efficacy Scale‐International, 16−64 (higher = greater perceived difficulty).

### 3.1 Patient‐reported and performance measures of gait

In relation to the five gait domains (Pace; Rhythm; Variability; Asymmetry; and Postural control) assessed using the electronic walkway, Walk‐12G scores showed moderate correlation with the Pace domain variable Step velocity (*r *= −0.46, *p* = 0.001) and moderate/poor correlations with Step length (*r *= −0.36, *p* = 0.01) as well as Step time variability (*r *=* *0.32, *p* = 0.027). The negative correlations reflect that decreased Step velocity and Step length were associated with increased perceived difficulties (Table 2). Perceived walking difficulty correlated poorly with all other spatiotemporal gait parameters related to Rhythm and Postural control.

**Table 2:** Spearman's rho correlations between the Walk‐12G and performance‐based measures of walking

Spatiotemporal gait domains	People with PD (*n* = 49)			
	Mean (*SD*)	Range	Walk‐12 PD	
			rho	*p*
Pace				
Step velocity (m/s)	1.18 (0.19)	0.67−1.6	−0.46	0.001
Step length (m)	0.62 (0.09)	0.33 −0.85	−0.36	0.01
Rhythm				
Step time (ms)	527 (40)	406−640	0.14	0.349
Swing time (ms)	381 (32)	293−455	−0.05	0.683
Variability				
Step length variability (m)	0.025 (0.006)	0.02−0.04	0.03	0.803
Step time variability (ms)	18.4 (5.2)	10−31.5	0.32	0.027
Asymmetry				
Swing time asymmetry (ms)	11.0 (8.3)	0.99−32.9	0.15	0.320
Step time asymmetry (ms)	7.5 (6.9)	0.5−27	0.27	0.053
Postural control				
Step length asymmetry (m)	0.033 (0.025)	0.00−0.10	0.21	0.164
Step width (m)	0.07 (0.02)	0.01−0.12	−0.05	0.736
Habitual walking				
Steps per day, median (q1–q3)	3653 (1853, 5890)	215−12 569	−0.46	0.001
Brisk walking (min/day)^a^, median (q1–q3)	23.5 (5.4, 42.2)	0.9−94.3	−0.35	0.022

Notes. PD, Parkinson's disease; *SD*, standard deviation; m/s, meter/second; m meters; ms, millisecond.

^a^Mins/day spent walking at a speed > 1.05 m/s.

In relation to walking behavior assessed using accelerometry, Walk‐12G scores showed a statistically significant association (*r *= −0.46, *p* = 0.001) with mean steps per day and a weaker correlation with time spent in brisk walking (*r *= −0.35, *p* = 0.022). That is, more steps taken per day and the more time spent in brisk walking, respectively, were related to less perceived walking difficulties.

### 3.2 Perceived walking difficulties in different groups

**Figure 1:**
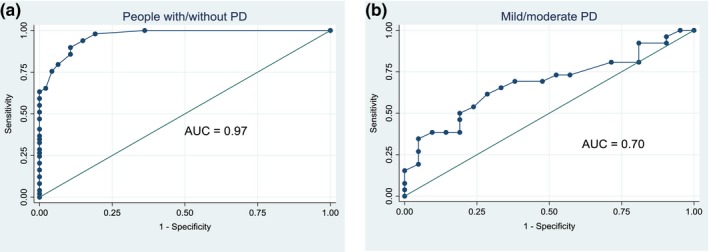
The receiver operating characteristic (ROC) curve with regard to the Walk‐12G's accuracy to distinguish between (a) people with and without Parkinson's disease and (b) people with mild and moderate disease severity (Hoehn and Yahr stages II/III, respectively)

People with PD reported significantly greater difficulties during walking than the healthy elderly group, with moderate–large effect size (ES = 0.82), see Table 3. The ROC analysis for these groups produced AUC values of 0.97 (Figure 1a), which indicates that the Walk‐12G had excellent capability to distinguish between people with PD and those without in our sample. Those at Hoehn and Yahr stage III reported significantly greater difficulties walking than people at the Hoehn and Yahr stage II (ES = 0.34) (Table 3). The area under the ROC curve for these two groups was 0.70 (Figure 1b), suggesting that the Walk‐12G has moderate capability to distinguish between disease stages. Analysis of the data upon removal of the two subjects who had undergone DBS surgery showed no significant differences in our findings (Supporting information Tables S1–S3).

**Table 3:** Between‐group differences of Walk‐12G for people with/without PD and at mild/moderate disease stages

	Median (q1–q3)	Range	Median (q1–q3)	Range	*p*	ES^a^
	PD (*n* = 49)		Controls (*n* = 47)			
Walk‐12G	12 (7, 20)	1–34	0 (0,1)	0–8	<0.001	0.82
	Mild (*n* = 22)		Moderate (*n* = 27)			
Walk‐12G	8.5 (6, 13)	1–25	15 (7, 23)	2–34	0.018	0.34

Notes. PD, Parkinson's disease.

^a^Effect size (ES), *r* = *Z*/√*N*.

## 4 DISCUSSION

The main finding of this study was that perceived walking difficulties in daily life appear to be most strongly associated with Step velocity—a laboratory‐assessed performance measure, and steps per day—a measure of walking behavior in free living among people with mild to moderate PD. Additionally, subgroup comparisons showed that the Walk‐12G has moderate ability to distinguish between mild–moderate PD stages and excellent ability to distinguish between people with and without the disease. These findings further support the clinical utility of the Walk‐12G in people with mild to moderate PD.

Walk‐12G scores correlated significantly with Step velocity, possibly reflecting that the latter is a robust parameter of walking behavior. Step velocity is a variable of the Pace domain which explains the highest proportion of total variance in a comprehensive PD gait model (Lord, Galna, Rochester et al., 2013). Gait speed, due to its robust nature, is therefore the recommended and most frequently used measure of walking performance in PD clinical trials (Lord, Galna, Rochester et al., 2013; Lord, Galna, Verghese et al., 2013). It is also possible that Step velocity, unlike other spatiotemporal domains captured in a controlled environment, is the most intuitive feature of walking and more likely therefore to influence perceived walking ability. Our observed moderate correlation with step velocity (*r *= −0.46), however, is weaker than that previously reported between the Walk‐12G and clinically assessed gait speed (*r *= −0.65) (Bladh et al., 2012). Disparities in testing protocols and disease duration between the samples (mean 13 years), compared to the current study (mean 6 years) may account for these observed differences (Sustakoski, Perera, VanSwearingen, Studenski, & Brach, 2015). Although similar investigations of the relationships between the Walk‐12G and performance‐based gait measures are lacking, fear of falling has been associated with slower gait speeds in healthy older adults (Maki, 1997) and people with PD (Bryant, Rintala, Hou, & Protas, 2014; Rochester et al., 2008). Additionally, Curtze et al. report that aspects of the pace domain were those most highly correlated with balance confidence, albeit when measurements occurred in the OFF phase of medication (Curtze, Nutt, Carlson‐Kuhta, Mancini, & Horak, 2016).

We observed a poor correlation between perceived walking and Step time variability, a gait feature altered at early disease stages, prior to detectable changes in gait speed (Baltadjieva, Giladi, Gruendlinger, Peretz, & Hausdorff, 2006). The literature concerning gait variability and fear of falling in PD—a separate patient‐reported construct largely explained by perceived walking difficulties—may be used for comparative purposes. A recent meta‐analysis reporting a weak relationship between fear of falling and variability parameters (Ayoubi, Launay, Annweiler, & Beauchet, 2015) also highlights the complexity of this relationship and how it may be confounded by both gait speed (Reelick, van Iersel, Kessels, & Rikkert, 2009) and previous falls (Ayoubi et al., 2013). Additionally, the clinometric properties of gait variability are not as firmly established as that of the pace domain and the use of variability measures to assess clinical training effects currently lacks efficacy (Galna, Lord, & Rochester, 2013; Lord, Galna, Rochester et al., 2013). Another factor for consideration in the interpretation of the results is that the specific items of the Walk‐12G focus less on walking features such as variability as they do factors such as speed, distance, and perceived effort.

We observed no correlation between perceived walking difficulties and aspects of gait Rhythm, Asymmetry, or Postural control. In comparison with previous studies of gait characteristics in PD (Galna et al., 2015), Asymmetry measures for our PD cohort were relatively low, which may explain these findings. It is also possible that the relatively low prevalence of freezing in our sample (only 10% of people reported freezing “often”), attenuated these relationships. In relation to the lack of correlation between aspects of Postural control and perceived walking, it is possible that our intermittent straight‐walking testing protocol, which did not measure turning while walking, limited the extent to which dynamic balance was challenged during walking. Mancini et al. used inertial sensors to continuously monitor aspects of turning in the home environment and reports strong correlations between turning velocity and the UPDRS motor score (Mancini et al., 2015).

We report a moderate correlation between the Walk‐12G and mean steps per day and time spent in brisk walking in free‐living environments. This is a previously unreported finding and provides further validity for the clinical use of the Walk‐12G in PD. The correlation we observed between walking behavior and the Walk‐12G must be viewed in relation to the literature where validity coefficients between patient‐reported and performance measures of physical activity levels range from weak to moderate at best (Helmerhorst, Brage, Warren, Besson, & Ekelund, 2012). It should also be noted that in the current study we are comparing separate constructs over different time periods—walking behavior over a 1‐week period compared with perceived walking difficulties during a period of 2 weeks.

The Walk‐12G is a patient‐reported outcome that assesses perceived walking difficulties during everyday life, which cannot be captured by performance‐based measures alone. It has previously been shown that the Walk‐12G does not solely reflect walking capacity, but is largely influenced by nonmotor factors such as self‐efficacy and depressive symptoms as well as self‐reported freezing of gait and fatigue (Kader, Ullen, Iwarsson, Odin, & Nilsson, 2017). This is a recognized attribute of patient‐reported as opposed to performance‐based measures among elderly with disability, whereby patient‐reported function can have stronger associations with psychosocial factors than with physical function (Bean, Olveczky, Kiely, LaRose, & Jette, 2011). The combination of performance‐based and patient‐reported outcomes of walking is therefore necessary to enable comprehensive assessment. Our findings concerning the ability of the Walk‐12G to distinguish between the subgroups PD and non‐PD as well as mild–moderate disease stages, are in line with a previous investigation of the difference in objectively measured gait abilities between these subgroups (Lofgren, Benka Wallen, Sorjonen, Conradsson, & Franzen, 2017). These results can be interpreted as providing further evidence for the clinical applicability of this scale.

## 5 LIMITATIONS AND FUTURE PERSPECTIVES

The main limitation of this study was that the sample was based on a convenience sample of people who had participated in a RCT study that addressed gait and balance problems. Moreover, only those with H & Yahr stages II–III were included. These aspects affect the external validity of the findings, which therefore need to be confirmed in future studies. Additionally, although the PD sample size of 49 is acceptable for gait laboratory data, the sizes of the subgroups of disease stages were small which limits the conclusions that can be drawn. These analyses need to be enhanced using larger samples of people at PD stages I−IV. At last, although this correlational study can determine the strength of the associations’ study findings cannot indicate the nature of causality between patient‐reported and performance measure of walking.

## 6 CONCLUSIONS

The Walk‐12G is an easily administered questionnaire which can be quick to apply in the clinical context to capture patient perspectives, for example, in the initial screening of walking among people with PD, prior to therapy. By focusing specifically on walking situations, Walk‐12G scores provide the opportunity for healthcare professionals to plan task‐specific training in line with patients’ needs. Our findings indicate how features of walking, such as Asymmetry and Rhythm, although reported to explain equal variance in gait models, are poorly reflected in patient‐reported outcome measures. This study provides evidence for the relationship between the Walk‐12G and both objectively measured walking pace and behavior in controlled and free‐living conditions, respectively. These findings indicate that patient‐centered training interventions should primarily address these gait aspects if they are also affected patient‐perceived walking difficulties in daily life.

## ACKNOWLEDGMENTS

The authors acknowledge all subjects who participated in this study. This study was funded by “Vårdalstiftelsen”, The Swedish Research Council for Health, Working Life and Welfare (FORTE), The Swedish Research Council, and The Swedish Parkinson Foundation.

## CONFLICT OF INTERESTS

The authors have no conflict of interest to report.

## References

[brb31081-bib-0001] Allen, N. E. , Schwarzel, A. K. , & Canning, C. G. (2013). Recurrent falls in Parkinson's disease: A systematic review. Parkinsons Disease, 2013, 906274 10.1155/2013/906274 PMC3606768 23533953

[brb31081-bib-0002] Ayoubi, F. , Launay, C. P. , Annweiler, C. , & Beauchet, O. (2015). Fear of falling and gait variability in older adults: A systematic review and meta‐analysis. Journal of the American Medical Directors Association, 16(1), 14–19. 10.1016/j.jamda.2014.06.020 25230892

[brb31081-bib-0003] Ayoubi, F. , Launay, C. , Annweiler, C. , Fantino, B. , Kabeshova, A. , & Beauchet, O. (2013). Fear of falling, falls, and gait variability in older community‐dwelling individuals: Is there an association? Journal of the American Geriatrics Society, 61(7), 1236–1238. 10.1111/jgs.12350 23855862

[brb31081-bib-0004] Baltadjieva, R. , Giladi, N. , Gruendlinger, L. , Peretz, C. , & Hausdorff, J. M. (2006). Marked alterations in the gait timing and rhythmicity of patients with de novo Parkinson's disease. European Journal of Neuroscience, 24(6), 1815–1820. 10.1111/j.1460-9568.2006.05033.x 17004944

[brb31081-bib-0005] Bean, J. F. , Olveczky, D. D. , Kiely, D. K. , LaRose, S. I. , & Jette, A. M. (2011). Performance‐based versus patient‐reported physical function: What are the underlying predictors? Physical Therapy, 91(12), 1804–1811. 10.2522/ptj.20100417 22003163 PMC3229045

[brb31081-bib-0006] Bilney, B. , Morris, M. , & Webster, K. (2003). Concurrent related validity of the GAITRite walkway system for quantification of the spatial and temporal parameters of gait. Gait & Posture, 17(1), 68–74. 12535728 10.1016/s0966-6362(02)00053-x

[brb31081-bib-0007] Bladh, S. , Nilsson, M. H. , Hariz, G. M. , Westergren, A. , Hobart, J. , & Hagell, P. (2012). Psychometric performance of a generic walking scale (Walk‐12G) in multiple sclerosis and Parkinson's disease. Journal of Neurology, 259(4), 729–738. 10.1007/s00415-011-6254-z 21956376

[brb31081-bib-0008] Bryant, M. S. , Rintala, D. H. , Hou, J. G. , & Protas, E. J. (2014). Influence of fear of falling on gait and balance in Parkinson's disease. Disability and Rehabilitation, 36(9), 744–748. 10.3109/09638288.2013.814722 23875814 PMC4167402

[brb31081-bib-0009] Canning, C. G. , Paul, S. S. , & Nieuwboer, A. (2014). Prevention of falls in Parkinson's disease: A review of fall risk factors and the role of physical interventions. Neurodegenerative Disease Management, 4(3), 203–221. 10.2217/nmt.14.22 25095816

[brb31081-bib-0010] Cohen, J. (1988). Statistical power analysis for the behavioral sciences (2nd ed.). Hillsdale, NJ: Lawrence Erlbaum.

[brb31081-bib-0011] Curtze, C. , Nutt, J. G. , Carlson‐Kuhta, P. , Mancini, M. , & Horak, F. B. (2016). Objective gait and balance impairments relate to balance confidence and perceived mobility in people with Parkinson disease. Physical Therapy, 96(11), 1734–1743. 10.2522/ptj.20150662 27149959 PMC5088223

[brb31081-bib-0012] Fritz, C. O. , Morris, P. E. , & Richler, J. J. (2012). Effect size estimates: Current use, calculations, and interpretation. Journal of Experimental Psychology: General, 141(1), 2–18. 10.1037/a0024338 21823805

[brb31081-bib-0013] Galna, B. , Lord, S. , Burn, D. J. , & Rochester, L. (2015). Progression of gait dysfunction in incident Parkinson's disease: Impact of medication and phenotype. Movement Disorders, 30(3), 359–367. 10.1002/mds.26110 25546558

[brb31081-bib-0014] Galna, B. , Lord, S. , & Rochester, L. (2013). Is gait variability reliable in older adults and Parkinson's disease? Towards an optimal testing protocol. Gait & Posture, 37(4), 580–585. 10.1016/j.gaitpost.2012.09.025 23103242

[brb31081-bib-0015] Godinho, C. , Domingos, J. , Cunha, G. , Santos, A. T. , Fernandes, R. M. , Abreu, D. , … Ferreira, J. J. (2016). A systematic review of the characteristics and validity of monitoring technologies to assess Parkinson's disease. Journal of NeuroEngineering and Rehabilitation, 13, 24 10.1186/s12984-016-0136-7 26969628 PMC4788909

[brb31081-bib-0016] Hammarlund, C. S. , Andersson, K. , Andersson, M. , Nilsson, M. H. , & Hagell, P. (2014). The significance of walking from the perspective of people with Parkinson's disease. Journal of Parkinson's Disease, 4(4), 657–663. 10.3233/JPD-140399 25147140

[brb31081-bib-0017] Hanley, J. A. , & McNeil, B. J. (1982). The meaning and use of the area under a receiver operating characteristic (ROC) curve. Radiology, 143(1), 29–36. 10.1148/radiology.143.1.7063747 7063747

[brb31081-bib-0018] Hausdorff, J. M. (2005). Gait variability: Methods, modeling and meaning. Journal of NeuroEngineering and Rehabilitation, 2, 19 10.1186/1743-0003-2-19 16033650 PMC1185560

[brb31081-bib-0019] Hausdorff, J. M. (2007). Gait dynamics, fractals and falls: Finding meaning in the stride‐to‐stride fluctuations of human walking. Human Movement Science, 26(4), 555–589. 10.1016/j.humov.2007.05.003 17618701 PMC2267927

[brb31081-bib-0020] Hausdorff, J. M. (2009). Gait dynamics in Parkinson's disease: Common and distinct behavior among stride length, gait variability, and fractal‐like scaling. Chaos, 19(2), 026113 10.1063/1.3147408 19566273 PMC2719464

[brb31081-bib-0021] Helmerhorst, H. J. , Brage, S. , Warren, J. , Besson, H. , & Ekelund, U. (2012). A systematic review of reliability and objective criterion‐related validity of physical activity questionnaires. International Journal of Behavioral Nutrition and Physical Activity, 9, 103 10.1186/1479-5868-9-103 22938557 PMC3492158

[brb31081-bib-0022] Hobart, J. C. , Riazi, A. , Lamping, D. L. , Fitzpatrick, R. , & Thompson, A. J. (2003). Measuring the impact of MS on walking ability: The 12‐Item MS Walking Scale (MSWS‐12). Neurology, 60(1), 31–36. 12525714 10.1212/wnl.60.1.31

[brb31081-bib-0023] Holland, A. , O'Connor, R. J. , Thompson, A. J. , Playford, E. D. , & Hobart, J. C. (2006). Talking the talk on walking the walk: A 12‐item generic walking scale suitable for neurological conditions? Journal of Neurology, 253(12), 1594–1602. 10.1007/s00415-006-0272-2 16924398

[brb31081-bib-0024] Kader, M. , Ullen, S. , Iwarsson, S. , Odin, P. , & Nilsson, M. H. (2017). Factors contributing to perceived walking difficulties in people with Parkinson's disease. Journal of Parkinson's Disease, 7(2), 397–407. 10.3233/JPD-161034 28505982

[brb31081-bib-0025] Lofgren, N. , Benka Wallen, M. , Sorjonen, K. , Conradsson, D. , & Franzen, E. (2017). Investigating the Mini‐BESTest's construct validity in elderly with Parkinson's disease. Acta Neurologica Scandinavica, 135(6), 614–621. 10.1111/ane.12640 27417912

[brb31081-bib-0026] Lord, S. , Galna, B. , & Rochester, L. (2013). Moving forward on gait measurement: Toward a more refined approach. Movement Disorders, 28(11), 1534–1543. 10.1002/mds.25545 24132841

[brb31081-bib-0027] Lord, S. , Galna, B. , Verghese, J. , Coleman, S. , Burn, D. , & Rochester, L. (2013). Independent domains of gait in older adults and associated motor and nonmotor attributes: Validation of a factor analysis approach. Journals of Gerontology. Series A, Biological Sciences and Medical Sciences, 68(7), 820–827. 10.1093/gerona/gls255 23250001

[brb31081-bib-0028] Lord, S. , Godfrey, A. , Galna, B. , Mhiripiri, D. , Burn, D. , & Rochester, L. (2013). Ambulatory activity in incident Parkinson's: More than meets the eye? Journal of Neurology, 260(12), 2964–2972. 10.1007/s00415-013-7037-5 23900754

[brb31081-bib-0029] Mak, M. K. , Wong‐Yu, I. S. , Shen, X. , & Chung, C. L. (2017). Long‐term effects of exercise and physical therapy in people with Parkinson disease. Nature Reviews Neurology, 13(11), 689–703. 10.1038/nrneurol.2017.128 29027544

[brb31081-bib-0030] Maki, B. E. (1997). Gait changes in older adults: Predictors of falls or indicators of fear. Journal of the American Geriatrics Society, 45(3), 313–320. 9063277 10.1111/j.1532-5415.1997.tb00946.x

[brb31081-bib-0031] Mancini, M. , El‐Gohary, M. , Pearson, S. , McNames, J. , Schlueter, H. , Nutt, J. G. , … Horak, F. B. (2015). Continuous monitoring of turning in Parkinson's disease: Rehabilitation potential. Neuro Rehabilitation, 37(1), 3–10. 10.3233/NRE-151236 26409689 PMC4745985

[brb31081-bib-0032] Matthews, C. E. , Ainsworth, B. E. , Thompson, R. W. , & Bassett, D. R. Jr (2002). Sources of variance in daily physical activity levels as measured by an accelerometer. Medicine and Science in Sports and Exercise, 34(8), 1376–1381. 12165695 10.1097/00005768-200208000-00021

[brb31081-bib-0033] Matthews, C. E. , Hagstromer, M. , Pober, D. M. , & Bowles, H. R. (2012). Best practices for using physical activity monitors in population‐based research. Medicine and Science in Sports and Exercise, 44(1 Suppl 1), S68–S76. 10.1249/MSS.0b013e3182399e5b 22157777 PMC3543867

[brb31081-bib-0034] Nero, H. , Benka Wallen, M. , Franzen, E. , Stahle, A. , & Hagstromer, M. (2015). Accelerometer cut points for physical activity assessment of older adults with Parkinson's disease. PLoS One, 10(9), e0135899 10.1371/journal.pone.0135899 26332765 PMC4558005

[brb31081-bib-0035] Peterson, D. S. , & Horak, F. B. (2016). Neural control of walking in people with Parkinsonism. Physiology (Bethesda), 31(2), 95–107. 10.1152/physiol.00034.2015 26889015 PMC4888974

[brb31081-bib-0036] Reelick, M. F. , van Iersel, M. B. , Kessels, R. P. , & Rikkert, M. G. (2009). The influence of fear of falling on gait and balance in older people. Age and Ageing, 38(4), 435–440. 10.1093/ageing/afp066 19451658

[brb31081-bib-0037] Riffenburgh, R. (2012). Statistics in medicine (3rd ed.). London, UK: Academic press.

[brb31081-bib-0038] Rochester, L. , Nieuwboer, A. , Baker, K. , Hetherington, V. , Willems, A. M. , Kwakkel, G. , … Jones, D. (2008). Walking speed during single and dual tasks in Parkinson's disease: Which characteristics are important? Movement Disorders, 23(16), 2312–2318. 10.1002/mds.22219 18816800

[brb31081-bib-0039] Sasaki, J. E. , John, D. , & Freedson, P. S. (2011). Validation and comparison of ActiGraph activity monitors. Journal of Science and Medicine in Sport, 14(5), 411–416. 10.1016/j.jsams.2011.04.003 21616714

[brb31081-bib-0040] Smulders, K. , Esselink, R. A. , Cools, R. , & Bloem, B. R. (2014). Trait impulsivity is associated with the risk of falls in Parkinson's disease. PLoS One, 9(3), e91190 10.1371/journal.pone.0091190 24608747 PMC3946755

[brb31081-bib-0041] Sustakoski, A. , Perera, S. , VanSwearingen, J. M. , Studenski, S. A. , & Brach, J. S. (2015). The impact of testing protocol on recorded gait speed. Gait & Posture, 41(1), 329–331. 10.1016/j.gaitpost.2014.10.020 25468684 PMC4271538

[brb31081-bib-0042] Swets, J. A. (1988). Measuring the accuracy of diagnostic systems. Science, 240(4857), 1285–1293. 3287615 10.1126/science.3287615

[brb31081-bib-0043] van der Eijk, M. , Nijhuis, F. A. , Faber, M. J. , & Bloem, B. R. (2013). Moving from physician‐centered care towards patient‐centered care for Parkinson's disease patients. Parkinsonism & Related Disorders, 19(11), 923–927. 10.1016/j.parkreldis.2013.04.022 23742970

[brb31081-bib-0044] van Nimwegen, M. , Speelman, A. D. , Hofman‐van Rossum, E. J. , Overeem, S. , Deeg, D. J. , Borm, G. F. , … Munneke, M. (2011). Physical inactivity in Parkinson's disease. Journal of Neurology, 258(12), 2214–2221. 10.1007/s00415-011-6097-7 21614433 PMC3225631

[brb31081-bib-0045] Walton, M. K. , Powers, J. H. III , Hobart, J. , Patrick, D. , Marquis, P. , Vamvakas, S. , … Burke, L. B. (2015). Clinical outcome assessments: Conceptual foundation‐Report of the ISPOR clinical outcomes assessment ‐ Emerging good practices for outcomes research task force. Value Health, 18(6), 741–752. 10.1016/j.jval.2015.08.006 26409600 PMC4610138

