# Supplementary material for: Patient‐reported and performance‐based measures of walking in mild–moderate Parkinson's disease
Source: Brain Behav. 2018 Aug 22;8(9):e01081. doi: 10.1002/brb3.1081 (PMC6160642; doi:10.1002/brb3.1081)
Supplement: Supplementary file 1 [file BRB3-8-e01081-s001.docx]

Supplementary Tables

**Table 1.** Characteristics of all participants, n=94

|  | **People with PD (n=47)** | | **People without PD (n=47)** | |
| --- | --- | --- | --- | --- |
|  |  | |  | |
| **Demographics** | **Mean (SD)** ^1^ | **Range** | **Mean (SD)** | **Range** |
| Sex (Female), n (%) | 28 (50.9) |  | 20 (42.5) |  |
| Age (year) | 76 (5.8) | 63−89 | 71 (6) | 60−88 |
| Body Mass Index | 25.8 (3.5) | 17.6−32.9 | 23.9 (2.3) | 19.6−29.6 |
| Years with PD, median (q1-q3)^2^ | 5.5 (2.5−9) | 1.5−26 | − | − |
| Hoehn & Yahr stage^3^  II, n (%) | II) 21 (45) | |  |  |
| III, n (%) | III) 26 (55) | |  |  |
| MMSE,^4^ median (q1-q3) | 28 (27−29) | 24−30 | 29 (27−29) | 25−30 |
| GDS,^5^ median (q1-q3) | 3 (1−6) | 0−12 | 1 (0−2) | 0−7 |
|  |  |  |  |  |
| **Mobility** |  |  |  |  |
| Walking aid indoors, n (%) | 4 (8) | − | 0 | − |
| Walking aid outdoors, n (%) | 19 (40) | − | 2 (4.3) | − |
|  |  |  |  |  |
| UPDRS-Motor (Part III)^6^ | 40 (10.9) | 12−67 | − | − |
| Physical functioning,^7^ median (q1-q3) | 65 (45−80) | 5−95 | 29 (28−30) | 0−30 |
| Freezing during walking^8^, n (%) |  |  |  |  |
| Never/seldom | 39 (79.6) |  |  |  |
| sometimes | 5 (10.2) |  |  |  |
| Often | 5 (10.2) |  |  |  |
|  |  |  |  |  |
| **Balance and falls** |  |  |  |  |
| MiniBESTest^9^ | 19.8 (4.0) | 10−27 | 22.8 (2.6) | 16−27 |
| Falls previous 12-months, n (%) | 24 (47) |  | 8 (17) |  |
| Falls Efficacy Scale International^10^ | 28.0 (8.5) | 16−48 | 17.9 (2.1) | 16−24 |
|  |  |  |  |  |
| **Patient-reported walking difficulties** |  |  |  |  |
| Walk 12-G, median (q1-q3) | 12 (7-21) |  |  |  |
|  |  |  |  |  |
| Daily Levodopa equivalent dose (mg) | 645 (315) | 120−1846 | − | − |
|  |  |  |  |  |
|  |  |  |  |  |

PD, Parkinson’s Disease, ^1^SD, Standard deviation Unless, otherwise stated

^2^ q1-q3, 25th−75^th^ percentilet

^3^ Stages I-V of disease progression (I=minimal disability, V=confined to bed/wheelchair)

^4^ Mini-Mental State examination, 0−30 (higher score=greater impairment)

^5^ Geriatric Depression Scale, 0-20 (higher score= greater likelihood of depression)

^6^ Motor examination of the Unified Parkinson’s Disease rating scale, 0-108 (higher score=greater severity)

^7^ Physical functioning scale of the SF-36, 0−100 (higher score=lesser severity)

^8^ Item 14 of the Unified Parkinson’s Disease rating scale- Activities of daily living (UPDRS-ADL)

^9^Mini balance evaluation systems test, 0-28 (higher score=better balance)

^10^ Falls efficacy Scale-International, 16−64 (higher=greater perceived difficulty)

**Supplementary Table 2.** Spearman’s rho correlations between the Walk-12G and performance-based measures of walking

|  | **People with PD (n=47)** | | | |
| --- | --- | --- | --- | --- |
|  |  |  |  |  |
|  |  |  | **Walk-12 PD** | |
| **Spatiotemporal gait domains** | **Mean (SD)** | **Range** | **rho** | **P** |
| *Pace* |  |  |  |  |
| Step Velocity (m/s) | 1.17 (.18) | .67−1.6 | -0.46 | 0.001 |
| Step Length (m) | .61 (.09) | .33 −.85 | -0.34 | 0.018 |
|  |  |  |  |  |
| *Rhythm* |  |  |  |  |
| Step time (ms) | 527 (40) | 406−640 | 0.20 | 0.169 |
| Swing time (ms) | 379 (31) | 293−455 | -0.02 | 0.894 |
|  |  |  |  |  |
| *Variability* |  |  |  |  |
| Step length variability (m) | .024 (.006) | .02−.04 | -0.01 | 0.952 |
| Step time variability (ms) | 18.5 (5.2) | 10−31.5 | 0.28 | 0.056 |
|  |  |  |  |  |
| *Asymmetry* |  |  |  |  |
| Swing time Asymmetry (ms) | 10.6 (8.1) | .99−32.9 | 0.09 | 0.530 |
| Step time Asymmetry (ms) | 7.3 (6.7) | .5−27 | 0.27 | 0.053 |
|  |  |  |  |  |
| *Postural control* |  |  |  |  |
| Step length asymmetry (m) | .033 (.025) | .00−.10 | 0.19 | 0.193 |
| Step width (m) | .07 (.02) | .01−.12 | -0.09 | 0.561 |
|  |  |  |  |  |
| **Habitual walking** |  |  |  |  |
| Steps per day, median (q1-q3) | 3094 (1796, 5720) | 215−12 569 | -0.45 | 0.002 |
| Brisk walking (min/day)^1^, median  (q1-q3) | 17.1 (5.4, 41.2) | .9−94.3 | -0.33 | 0.036 |
|  |  |  |  |  |

Abbreviations:

PD, Parkinson’s Disease; SD, Standard deviation, m/s; meter/second, m; meters, ms; millisecond

^1^Mins/day spent walking at a speed> 1.05 meters/second

**Supplementary Table 3**. Between group differences of Walk-12G for people with/without PD and at mild/moderate disease stages.

|  | **Median (q1-q3)** | **Range** | **Median (q1-q3)** | **Range** | **p** | **ES^1^** |
| --- | --- | --- | --- | --- | --- | --- |
|  |  |  |  |  |  |  |
|  | **PD**  (n=47) |  | **Controls**  (n=47) |  |  |  |
| **Walk-12G** | 12 (7, 21) | 1-34 | 0 (0,1) | 0-8 | <.001 | .82 |
|  |  |  |  |  |  |  |
|  | **Mild**  (n=21) |  | **Moderate**  (n=26) |  |  |  |
| **Walk-12G** | 9 (6, 13) | 1-25 | 15 (7, 23) | 2-34 | .035 | .31 |

PD, Parkinson’s Disease,

^1^ Effect size (ES), r=Z/√N
